# Supplementary figures and images for: CD11cHi monocyte-derived macrophages are a major cellular compartment infected by Mycobacterium tuberculosis
Source: PLoS Pathog. 2020 Jun 16;16(6):e1008621. doi: 10.1371/journal.ppat.1008621 (PMC7319360; doi:10.1371/journal.ppat.1008621)

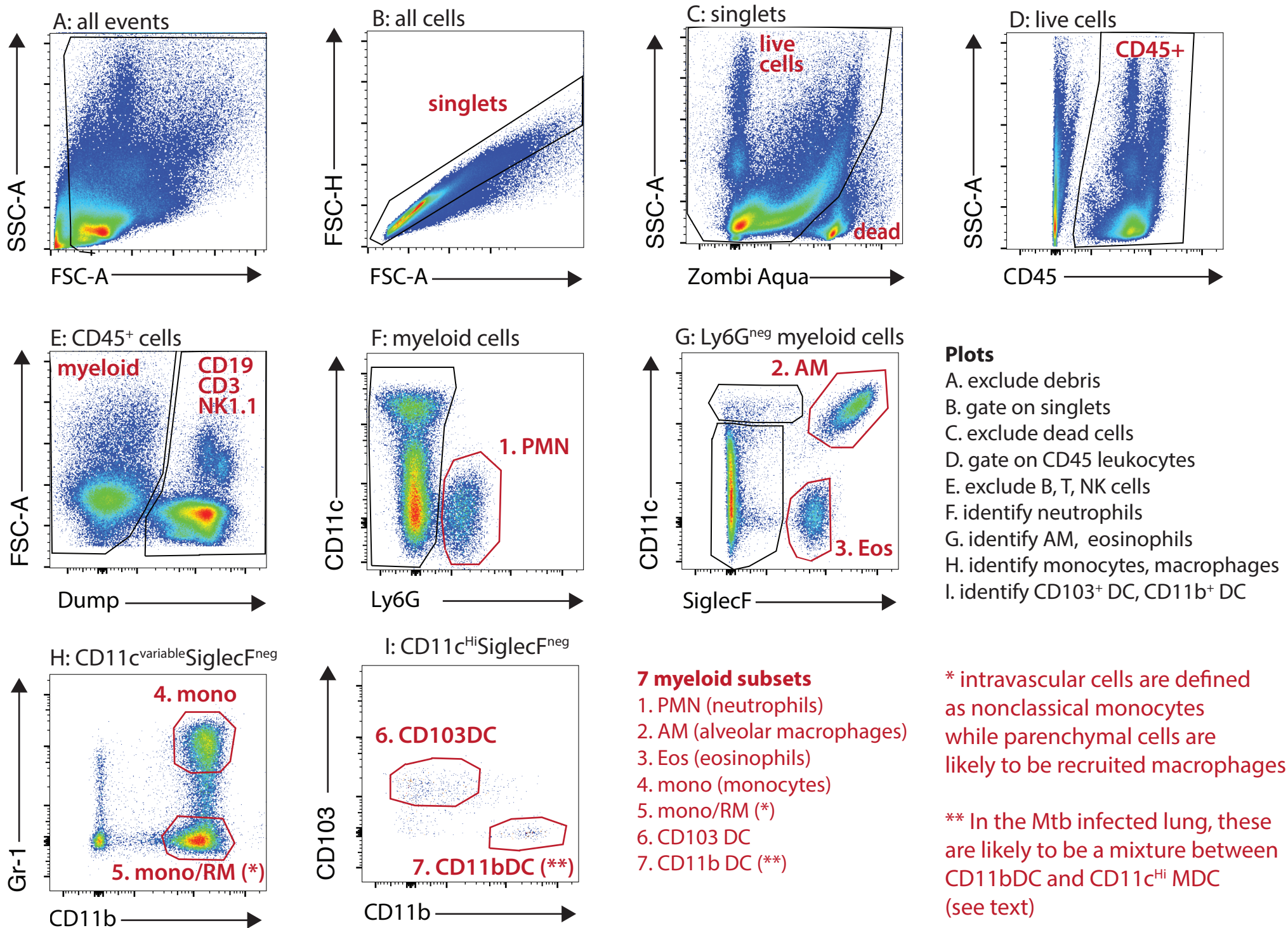

**Figure S1**

Supplement: S1 Fig — CD11b and CD11c are frequently used to distinguish alveolar macrophages (AM) from pulmonary dendritic cells (DC): AM are generally CD11bloCD11chiMHCIIlow-int, while DC are CD11bint-hiCD11chiMHCIIhi. This strategy works well for uninfected mice housed under conventional conditions. However, during inflammation, AM upregulate both CD11c and MHCII. Therefore, the following scheme takes advantage of SiglecF to identify AM, independently of MHCII. Single cell suspensions obtained from the lungs of mice were gated based on (a) scatter, (b) singlets, and then dead cells excluded (c). Leukocytes in the lung were identified based on CD45 staining (d) and lymphoid cells were excluded by using a dump channel that consisted of anti-Thy1, CD19, and NK1.1 (e). Neutrophils (PMNs, “1”) were identified based on Ly6g expression (f). AM (“2”) were identified by their siglecFHiCD11cHi phenotype, while eosinophils (Eos, “3”) expressed lower amounts of SiglecF and no CD11c (i.e., siglecFintCD11c-) (g). CD11cintCD11bHi cells were identified as recruited macrophages (RM, “5”) and monocytes, and monocytes were further distinguished by their Ly6C expression (“4”). Finally, the remaining CD11cHi cells were designated as DC, and 2 different subsets identified by their expression of CD103 (i.e., CD103DC or cDC1, “6”) or CD11b (i.e., CD11bDC or cDC2, “7”). In the Mtb infected lung, siglecF-CD11cHiCD11bHi cells are likely to be a mixture of CD11bDC and CD11cHi MDC. (PDF) [file ppat.1008621.s001.pdf]

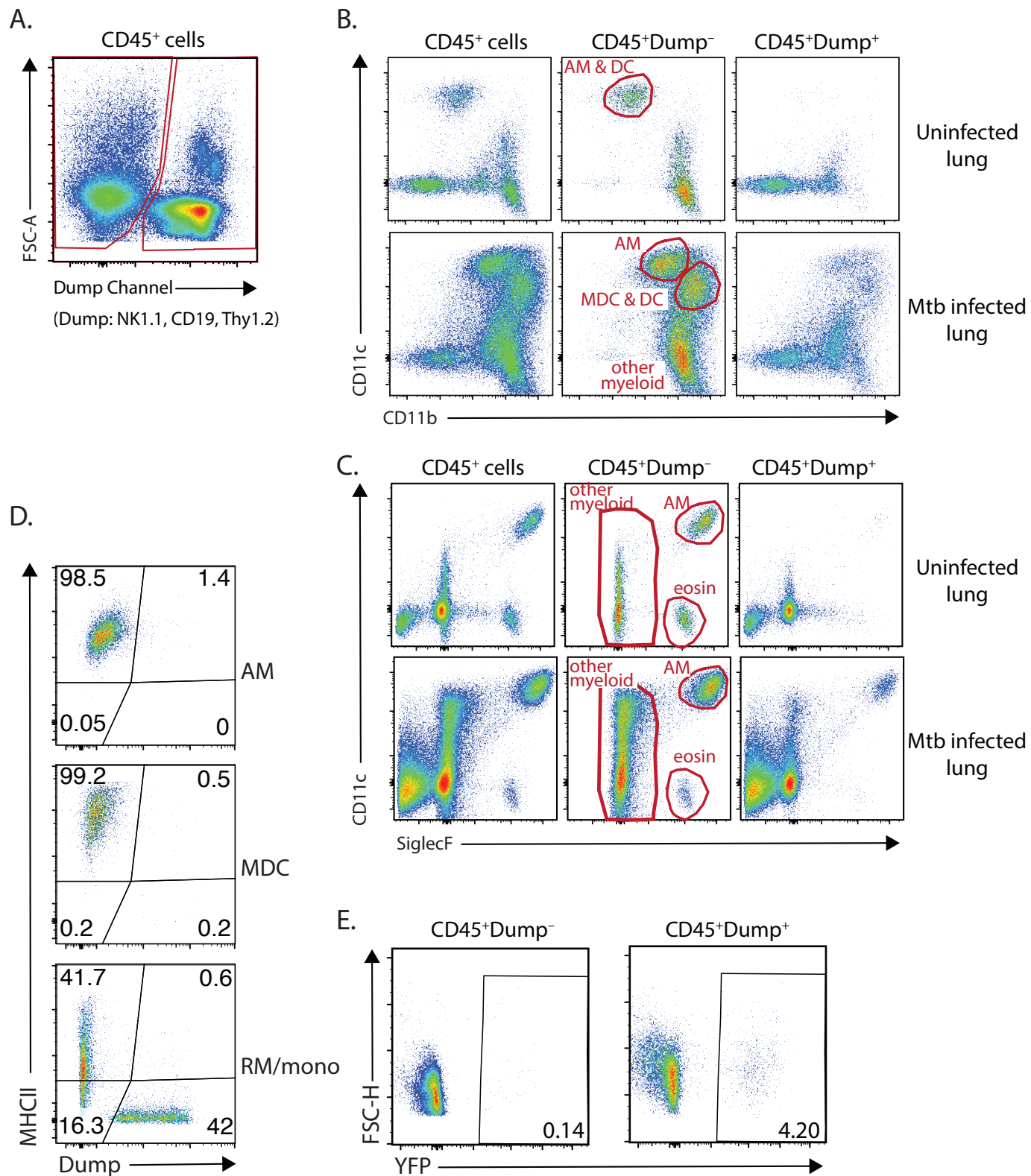

**Figure S2**

Supplement: S2 Fig — In addition to eliminating debris, doublets, and dead cells from our analysis, an essential part of our strategy was to exclude lymphoid cells. After gating on CD45+ cells, lymphoid cells were identified by using a “dump” gate consisting of a mixture of antibodies specific for Thy1, CD19, and NK1.1 (a). Although these markers primarily identify lymphoid cells, some lymphoid cells are known to express low levels of CD11b, CD11c, and SiglecF, particularly when activated, which could lead to misclassification of cell types. Although there are few Dump+ cells that express CD11b, CD11c, or SiglecF in the lungs of uninfected mice (b, c), one sees a more complicated pattern of CD11b and CD11c expression by CD45+ cells by cells isolated from the lungs of Mtb-infected mice (S2b, left), by CD45+dump- cells (center), or by lymphoid cells (right). This strategy is even important when CD11b, CD11c, and SiglecF staining are used to identify lung myeloid cells (c). Thus, our staining panel and gating strategy identify seven major populations of distinct myeloid cells that are present in the lungs, independently of M. tuberculosis infection. This strategy facilitates accurate enumeration of myeloid cells and measurement of phenotypic changes during infection, independent of the markers used to identify the different cell populations. A possible confounder is whether activated myeloid cells express Thy1, CD19, or NK1.1 lineage markers. Analysis of Mtb-infected RAG knockout mice, which lack B and T cells but still have NK cells, shows that few if any activated cells (i.e., MHCII+) in the AM or MDC gate express the “dump markers” (d). As for the RM population, there were “Dump+” cells, which were presumably NK cells, but none in the MHCII+ region. The cells show up in the RM gate as they lack expression of the other markers used to identify distinct myeloid populations (i.e., they lack SiglecF, CD11c, Ly6c, and MHCII). Additionally, we determined the percentage of cells in the dump ch [file ppat.1008621.s002.pdf]

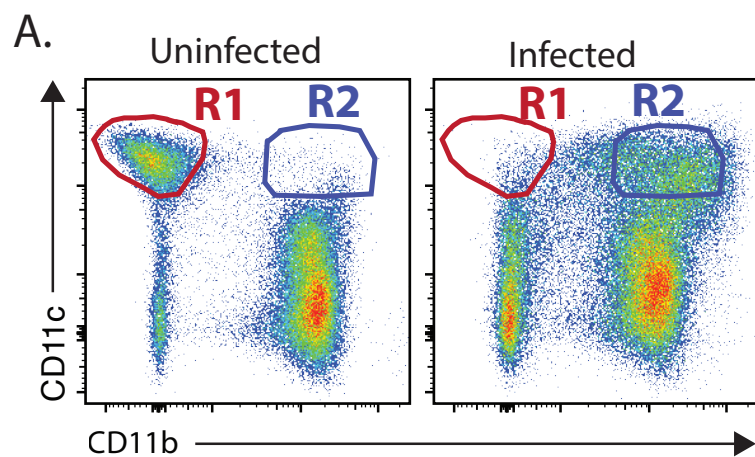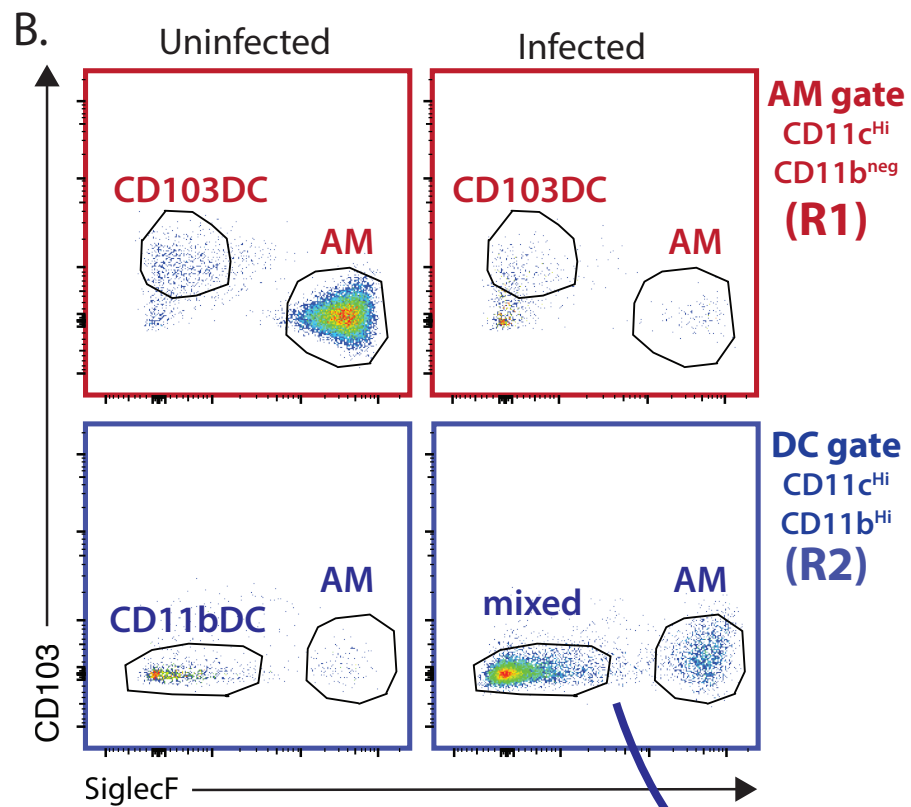

CD11bDC  
and  
CD11c<sup>Hi</sup> MDC

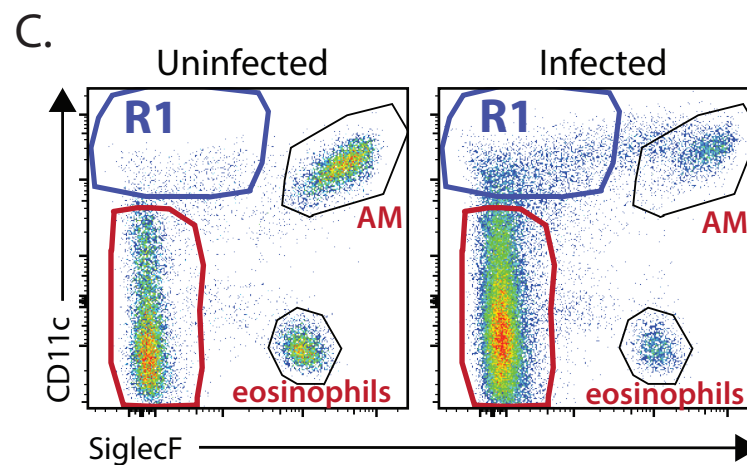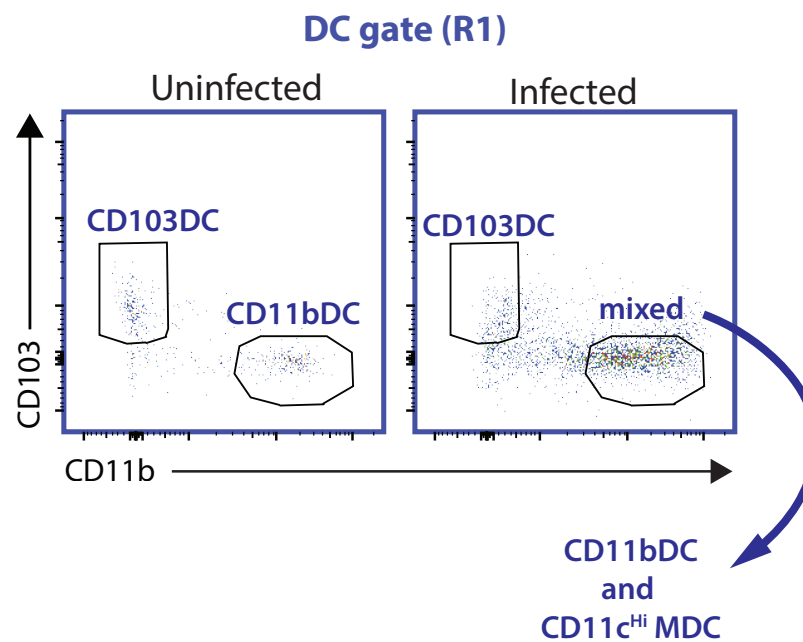

**Figure S3**

Supplement: S3 Fig — CD11b and CD11c can be used to discriminate AM and DC in uninfected mice; however, they need to be used in conjunction with other markers during Mtb infection. For example, in uninfected mice, CD11bloCD11chi cells are mostly AM (S3A Fig, left panel). Further analysis of the R1 gate shows that most of the cells are AM with some CD103DC (S3B Fig, top left panel). Similarly, CD11bhiCD11chi cells (S3A Fig, R2 gate) are nearly all CD11bDC (S3B Fig, bottom left panel). However, during Mtb infection, the pattern is more complex. The few CD11bloCD11chi cells (S3A Fig, R1 right panel) are a mixture of AM and DC (S3B Fig, R1), and the CD11bhiCD11chi cells are mostly CD11bDC and MDC but also AM (S3B Fig, R2). In contrast, using the gating scheme described above (S1 Fig), AM and eosinophils are unambiguously identified based on siglecF and CD11c, independently of infection (S3C Fig). The CD11chisiglecFlo cells define DC (Fig 3C, “DC gate”), while the CD11clow-intermsiglecFlo cells are RM and monocytes. The cells in the DC gate include both CD103DC and DC11bDC before infection, but after infection, CD11bDC dominate. As we discuss later, we think that although there are CD11bDC in this population, most of the cells are CD11chi monocyte-derived cells (CD11chi MDC). (PDF) [file ppat.1008621.s003.pdf]

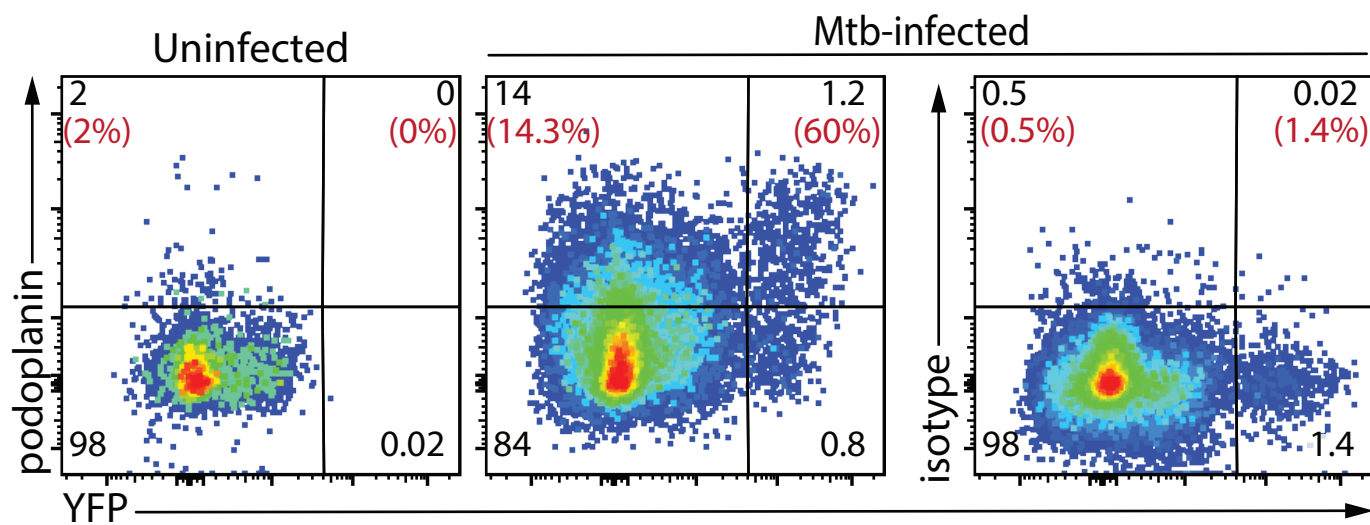

Figure S5

Supplement: S5 Fig — Lung cells were obtained from uninfected mice, or mice four weeks after aerosol infection with Rv.YFP. Podoplanin expression was analyzed by flow cytometry after gating on live CD45+ cells, excluding T cells, B cells, NK cells, and PMN. The black numbers are the percentage of events in each quadrant. The red numbers are the percentage of YFP-ve or YFP+ve events that express podoplanin. (PDF) [file ppat.1008621.s005.pdf]

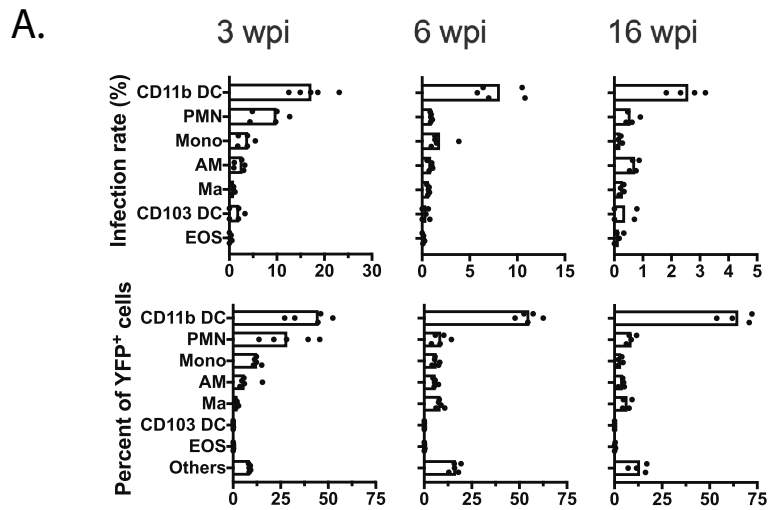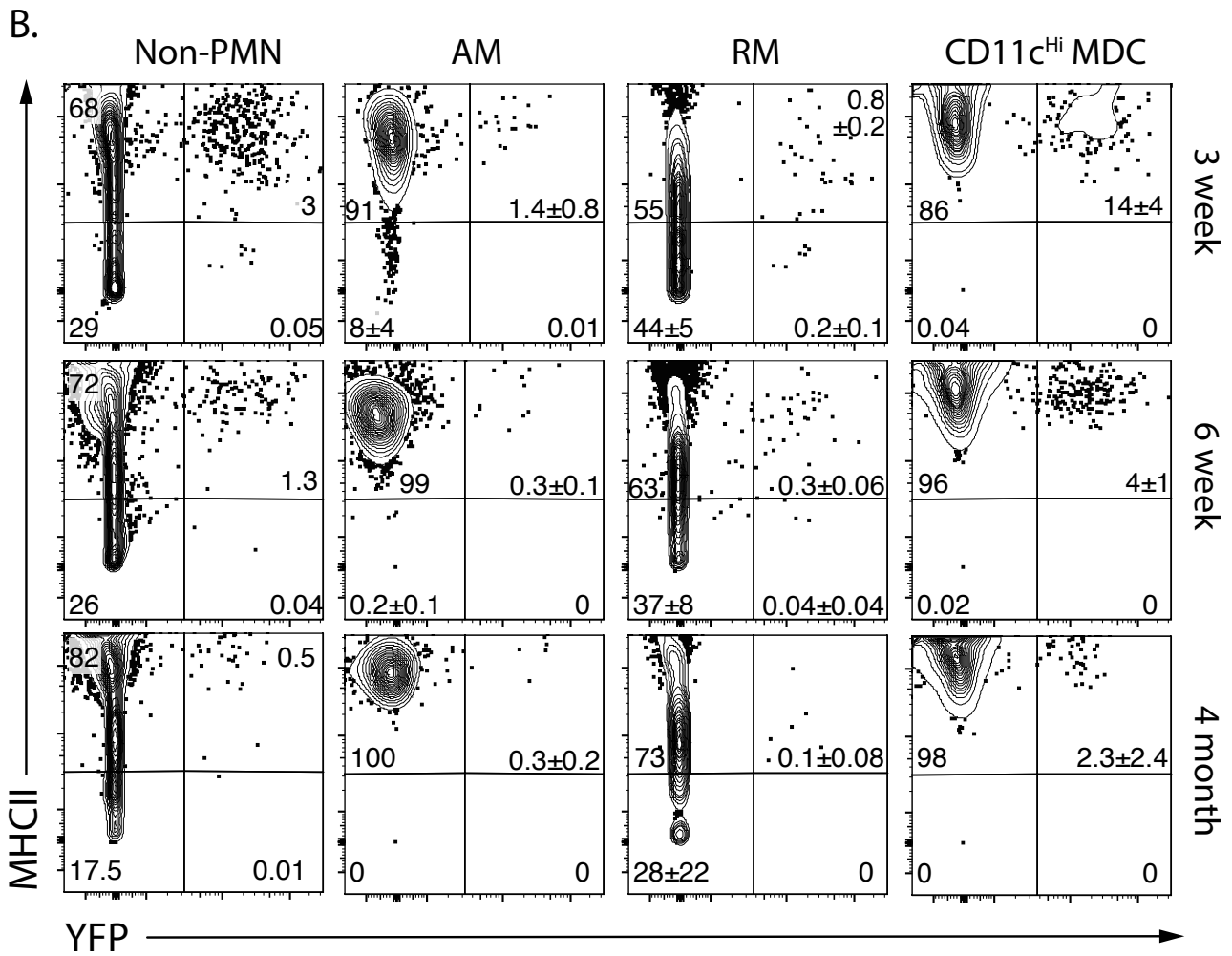

**Supplemental Figure 6**

Supplement: S6 Fig — The H37Rv.YFP strain we use was transformed using a plasmid that expresses sfYFP driven by a constitutive promoter. These plasmids are lost over time and at late timepoints (≥16 wpi) most infected cells no longer express the plasmid based on the lack of fluorescence of CFU grown from lung homogenates. Thus, we cannot rule out that there was preferential plasmid loss in certain cell types or differential degradation of YFP. However, myeloid cells could still be detected at late time points up till 9 months after infection. The distribution of YFP among different cell types (A) and their expression of MHC II (B) 4 months after infection follows a similar trend as observed at 3 and 6 wpi (see Figs 9 and 10). However, because of the issue of plasmid loss, one must interpret these data cautiously. (PDF) [file ppat.1008621.s006.pdf]
