# Supplementary material for: CD11cHi monocyte-derived macrophages are a major cellular compartment infected by Mycobacterium tuberculosis
Source: PLoS Pathog. 2020 Jun 16;16(6):e1008621. doi: 10.1371/journal.ppat.1008621 (PMC7319360; doi:10.1371/journal.ppat.1008621)
Supplement: S4 Fig — A. Using well-described transcriptional gene signatures for AM, macrophages, DC and monocytes [2, 32], YFPpos AM, CD11cHi MDC, and RM were compared to well-defined lung myeloid cell populations from uninfected mice (data from www.Immgen.org) using a heat map with global normalization. The YFPpos AM have a transcriptional profile matching AM, while YFPpos CD11cHi MDC and resemble the monocyte/macrophage lineage. B. We developed a 21 gene signature (here, referred to as “ImmGen 1”) that distinguishes ImmGen DC from macrophages. An analysis using the expression of the 15 genes that are preferentially expressed in macrophages (top), the 6 genes that are preferentially expressed in DC (middle), or the combined 21 gene signature is quantified for 43 myeloid unique ImmGen myeloid cell populations, grouped by cell type. Red bar, median. pDC, plasmacytoid DC. Blue, ImmGen lung populations. C. The list of genes that make up the 21 gene signature. (PDF) [file ppat.1008621.s004.pdf]

A.

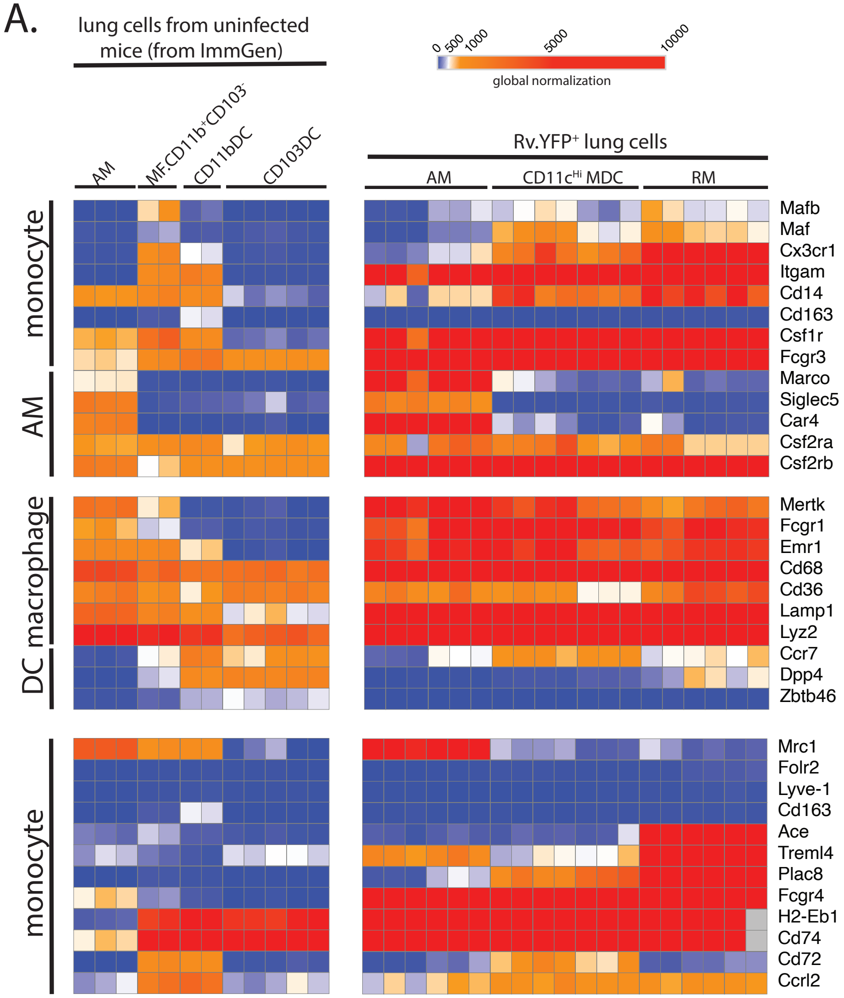

Signature	Ref
DC signature	[1]
macrophage signature	[2]
AM vs. monocytes	[3]
DC vs. macrophage	[3]
Monocyte subtypes	[4]

B.

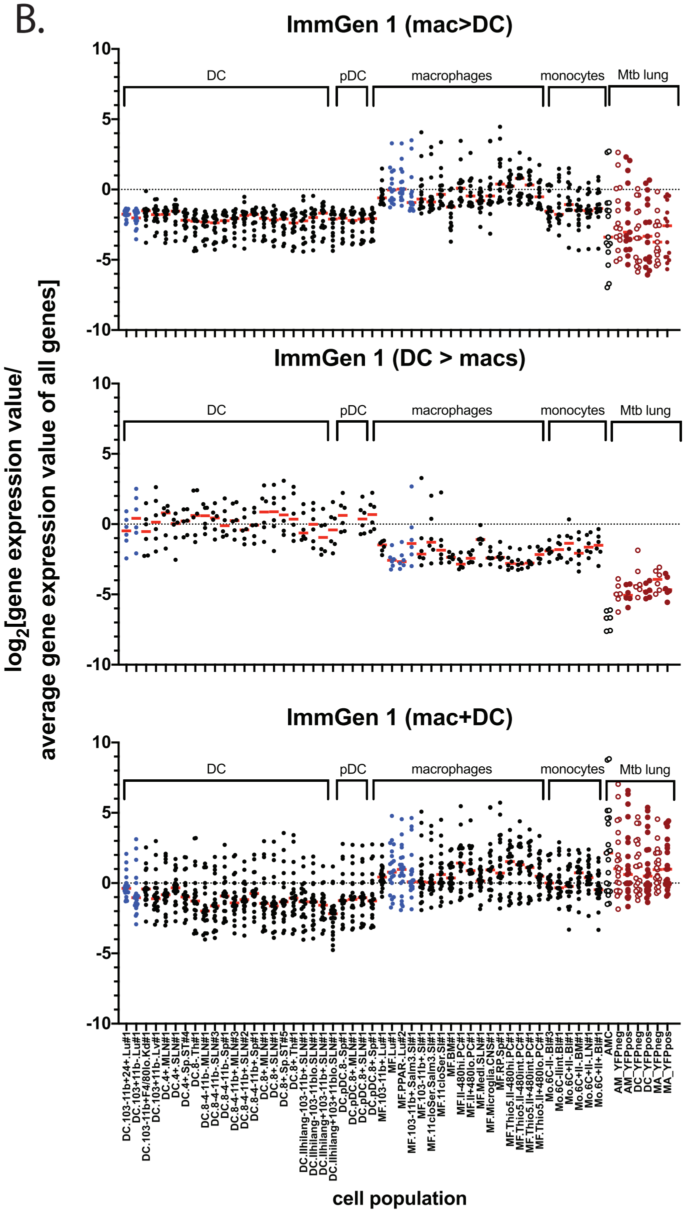

C.

macrophages>DC

Mertk
Tlr8
Fgd4
Tlr4
Ptgs1
Tmem195
Slc16a10
Mitf
Abcc5
Nfia
B3galtl
Tbc1d24
Sntb2
Zdhhc1
8430406I07Rik

DC>macrophages

Btla
Ap1s3
Amica1
Sema7a
Strbp
Usp53

Figure S4
