# Supplementary material for: CD11cHi monocyte-derived macrophages are a major cellular compartment infected by Mycobacterium tuberculosis
Source: PLoS Pathog. 2020 Jun 16;16(6):e1008621. doi: 10.1371/journal.ppat.1008621 (PMC7319360; doi:10.1371/journal.ppat.1008621)
Supplement: Attachment — Submitted filename: Point by point.docx [file ppat.1008621.s007.docx]

Reviewer's Responses to Questions

**Part I - Summary**

**Reviewer #1:**

The manuscript by Lee and colleagues titled “a subset of CD11chi monocyte-derived macrophages are the dominant niche for Mycobacterium tuberculosis” addresses a rapidly evolving and highly significant area of tuberculosis research. As our appreciation of macrophage ontogeny and heterogeneity expands it is important to generate a functional appreciation of how the different macrophage subsets contribute to control or progression of tuberculosis in vivo. In this paper the authors describe a new subset of monocyte derived macrophages that are defined by the surface marker profile CD11b+CD11c+ MHCIIhi and characterized by additional transcriptional profiling. When they examined the distribution of YFP-expressing Mtb in the different macrophage populations they found that these cells contained an unusually high burden of bacteria. On the basis of these observations they refer to these cells as the “dominant niche” for Mtb in the mouse lung (9 times in the text).

I find the data presented in the paper to be of high quality and they are undoubtedly of value to the field at this particular time. However, I think the repeated use of the term “dominant niche”, which implies functional significance beyond mere abundance, to be a considerable exaggeration of what has actually been demonstrated.

1. There is no information on the status of the bacteria in these cells, and they could be fully viable and replicative, or fully under host control.

2. There is no manipulation of this host cell population to demonstrate that they play any role of significance to either disease control or progression. To be able to claim that they are the “dominant niche” one would expect to see some experimental validation.

Absent any functional information the claim that this is the “dominant niche occupied by M tuberculosis long-term” is conjecture. As this remains an unproven hypothesis it should be presented as such and not sold as proven fact. The paper by Huang and colleagues included functional manipulation of cell subsets and demonstrated impact on bacterial burden, and more recently the same group published Dual RNA-seq analysis showing the “matching” stress signatures of pathogen and host cell subset (Pisu et al. Cell Reports 2020). I fully accept that these papers were generated with data from a single early time point and that the conclusions and findings in these papers do not refute the current data in this manuscript, which emphasize later time points in infection progression and probes the granularity of monocyte-derived cell subsets. However, the earlier papers included fitness reporter bacterial strains and experimental manipulation of the cell subsets to demonstrate their functional significance in vivo and the current study lack any comparable data.

The data in the current paper opens the possibility to the presence of a permissive subset of recruited monocyte-derived cells that may have considerable significance post development of an acquired immune response. I agree this is exciting but on the basis of the data presented it is a possibility and not a fact, and its significance remains to be established experimentally.

We agree with the reviewer’s assertion that we have little experimental data to support the hypothesis that “TP cells” are a permissive niche for the Mtb and as pointed out by all reviewers, an important challenge faced by ourselves and the field in general, is the ability to ascertain the viability of Mtb in situ or ex vivo. Our use of the term “dominant niche,” was intended to convey an abundant cellular location occupied by the bacilli. We appreciate how the term “niche” may have implied a functional significance not explored in the context of our current paper. Please see below for a description of how we modified the manuscript in response to the reviewer’s comments.

**Reviewer #2:**

This manuscript by Lee et al examines the intracellular niche of M. tuberculosis (Mtb) during chronic infection. Although this topic has been studied extensively by several other groups, this work does advance new knowledge on the subject. That said, the paper could be written more clearly and in a more focused manner. I have several comments and questions which should be addressed.

Please see below for our specific responses to the reviewer’s queries.

**Reviewer #3:**

The ability to distinguish between different cells of the myeloid lineage and their ontogeny particularly macrophages has been a major challenge in the context of Mtb infection. Isolating discrete populations from the heterogenous macrophage populations remains a barrier. In the current submitted manuscript; Lee et al., identify using flow cytometric analysis a subset of CD11chi monocyte derived cells which have an increased presence in the lung post Mtb infection. Moreover, these cells seem to be induced or influenced by IFNγ as per their gene expression signature and evidence from the IFNγR KO mouse. In general, the manuscript has been prepared thoughtfully and is well written, particularly the discussion. Some minor additions and alterations are needed to fully explain and add clarity to readers on the conclusions of the data presented. The data presented in the study thus far is quite comprehensive and well supported.

Please see below for our specific responses to the reviewer’s queries.

**Part II – Major Issues: Key Experiments Required for Acceptance**

**Reviewer #1:**

If the authors wish to argue the functional significance of this cell subset as a niche that supports infection, they need to include data demonstrating functional impact. However, I would be perfectly satisfied by a more circumspect handling of the interpretation of the data. I think the hypothesis is important, our knowledge of lung macrophages is woefully incomplete, and these data are timely and of value to the field.

Based on the reviewer’s recommendation, we have modified the manuscript in the following ways:

- We have removed all uses of the term “dominant niche”
- We have avoided referring to the idea that these cells are potentially restrictive or permissive, except in the discussion where we present it as a hypothesis to test in the future

Our changes include;

- Removing all instances of the phrase “dominant niche” including
  - A new title for our paper and removal of the term “dominant niche”
  - Rewritten our abstract to remove the term “dominant niche” and propose the defining the niche of Mtb as a future goal
  - Removed “dominant niche” from the author summary
  - Removed the word “niche” except when articulating important questions for the field

**Reviewer #2:**

1) To me the most important finding of the paper is that the CD11c+CD11b+ myeloid population that is the predominant niche for Mtb during chronic infection, and has been referred to as dendritic cells in most of the TB literature for years are actually monocyte-derived cells that are most akin to macrophages (this is also the title of the manuscript). The best evidence for this would be the RNA-Seq analysis shown in Fig. S4, as the rest of the data does not conclusively make this point. Why is the most important figure shown only as a supplemental figure? The figure legend suggests this is old data that was performed using sorting “based on a limited number of cell surface markers.” If so, as this is the cornerstone of the manuscript, I believe this analysis should be repeated sorting cells using markers that they use throughout the manuscript and that they currently think is optimal. In addition, although the heat maps are helpful, is there a bioinformatic platform that could be utilized to better assess the relatedness between the various populations? I think this central message could be made more clearly and more rigorously. I think this analysis would be an important contribution to the field, but it needs to be done well and to be presented prominently in the paper.

We agree with the reviewer that this is an important point and we are concerned that it appears to be buried. We have taken his/her suggestion and put additional data into the main body of the figure (see below). In fact, we present additional bioinformatic analyses to support the conclusion. We split the heat maps and now include the heat map of the macrophage and DC signature in the NEW Fig.4. This figure also includes an analysis of these two signatures using a more quantitative approach and comparing the performance across all myeloid cell subset in the ImmGen database. Finally, we use a gene signature derived from ImmGen, which differentiates macrophages from DC, and perform a Pearson correlation analysis. This analysis shows that all of our sorted populations have a significantly higher correlation with the ImmGen macrophage populations than with the DC populations.

2) as shown by RNA-Seq (although this data is buried in Fig. S4). Although previous work by the authors and others has shown that at least some of these cells are derived from monocytes, several possibilities have remained: 1) that although some are monocyte derived, most are still conventional DCs that perhaps express some monocyte markers during the inflammatory milieu of TB, or 2) Most are monocyte-derived, but for all intensive purposes they differentiate into cells that are largely indistinguishable from DCs. Although it cannot be ruled out that a small subset of these cells may be true DCs, as the authors acknowledge, Fig S4 is the most convincing data to date that most of these are monocyte-derived cells that most resemble macrophages. This should be highlighted more and I believe this data should be moved to be one of the primary figures.

We have now moved Fig.S4 to the main body of the manuscript and renamed it Fig.4.

3) Instead of highlighting the above, the authors seem to spend excessive time (half of Figure 1 and again returning to in in Figure 9), refuting the designation of these cells as interstitial macrophages “IM”, which was a designation made largely in a single paper (Huang et al), and based largely on CD64 and MerTK staining. I believe the point could be made more simply and, again as a subpoint to the fact that these cells are not mostly DCs. The discrepancy in the % of the cells that are MerTK+ (the primary difference between this paper and that of Huang et al.) could perhaps be explained by the dose/route of infection (50-100 CFU by aerosol in this paper vs. 1000 CFU intranasally by Huang), but the biological significance of what % of the macrophages are MerTK+ is not clear and should be de-emphasized.

We appreciated that the reviewer may believe that we are making too much of the difference between the IM described by David Russell’s lab, and the CD11chighMDC described in our manuscript. We agree that some of the discrepancies in the study by Huang et al and our study arise from differences in the route, dose, and time point analyzed, which we consider in our discussion. However, the paper by Huang et al elegantly identify important differences between IM and AM and propose that AM are permissive while IM are restrictive. It is not our purpose to refute their designation of IM. There is an extensive literature on IMs in the lung, and considerable debate about their origins and their classification. Our interpretation, based on the literature, is that IMs are monocyte-derived cells. As the reviewer points out above, and mentioned in our manuscript, one cannot eliminate the possibility that there are mixed populations of cells when using bulk transcriptomic analysis. Our use of flow cytometry as a single cell analysis shows that based on CD64 and MertK expression, IM can be identified as a myeloid cell population in the lung, and we hypothesize that IM could be a precursor of CD11c^Hi^MDC. We believe these data are important both to establish the connection between the two studies, as many readers will want to know how the populations are related, and to understand how these populations evolve over time.

4) The manuscript uses several different designations throughout the manuscript in reference to these major infected cell type. Initially they are called *CD11bDCs and then switch at various times to calling them different names, including between “CD11c hi MC” and “MDC”. It is understandable that they initially call them CD11bDC as this is what they have been called historically, but the meaning of * is only in the figure legend and thus confusing. However, I believe they should settle then settle on the terminology they want to use and use it consistently thereafter.

We recognize this is a problem and apologize for the confusion. We sought to use CD11bDC only in first part of the paper, and then switch to CD11cHi MDC. We were not calling the cells “*CD11bDCs” but rather using the asterisk to qualify the meaning of CD11bDC in the figure so it wouldn’t be confusing! Obviously, we did not succeed. We have gone through the paper and tried to be more consistent in the text and in the figures.

5) In the introduction to Figure 2 on p.8, the authors state that “prior studies that identified cell types infected by Mtb using definitions based mostly on CD11b and CD11c, focused on one or two cell types, or looked at early timepoints” and cite several references for this statement. However, ref 9 (Cohen et al) is omitted in this section. Figure 1 in Cohen et al., used a panel of several markers, looked at several myeloid cell types, and arrived at the same nomenclature used here at similar timepoints. Thus, Figure 2 is consistent with and confirms this prior work and this should be acknowledged.

We have added the Cohen et al reference.

6) The authors use a dump gate of CD19, Thy1, and NK1.1 to exclude B cells, T cells, and NK cells from the myeloid populations they want to look at. These markers are usually lineage specific under non-inflamed conditions, but are the authors sure that these markers are not upregulated on myeloid cells during pulmonary TB? In Fig. S2 they argue that the dump is necessary because B cells/T cells/NK cells upregulate myeloid markers during Mtb infection. However, this same data could also be explained if myeloid cells upregulate CD19, Thy1, or NK1.1.

It is well-appreciated that CD11b, CD11c, and MHCII (the latter, demonstrated primarily from human T cells only) are upregulated on activated T cells. Among the 65 distinct populations of myeloid cells analyzed by ImmGen, only BM.MF express low levels of NK1.1; DC.II^hi^lang-103-11b^lo^.SLN, MF.480^int^.Lv.v2, and Mo.6C^+^II-.LN express medium levels of CD19; and, BM.MF, DC.LC.Sk, Mo.6C^+^II-.LN, and DC.8+.Th express medium levels of Thy1. Of course, these determinations are made by bulk transcriptomic analysis and the populations may be contaminated by small numbers of other cell types. Most importantly, although they are all non-activated cells, and their expression may not reflect what happens in the lung of an infected individual. However, these are all commonly used lineage markers.

To address this issue empirically, we determined the percentage of cells in the dump channel that were YFP+. Cells in the myeloid gate were ~30 times more likely to be infected than cells in the dump gate (~4% vs 0.14%). While these data do not directly address whether lymphoid lineage markers are expressed on myeloid cells, they do say that the dump channel is excluding very few infected cells from our analysis. We also addressed this question by using our flow panel to analyze Mtb-infected RAG knockout mice that lack B and T cells but still have NK cells. There were essentially no cells in the AM or MDC gate that express the “dump markers”. As for the RM population, there were “Dump+” cells, which were presumably NK cells, but none in the MHCII+ region. This is expected because there are essentially the cells that lack expression of all other markers (SiglecF, CD11b, CD11c, MHCII).

We’ve added the information to supplemental figure 2 and its legend.

7) In Fig. 3 the authors show that AM were activated by IFNg and on p. 9 they state, based on this data, that “we infer that T cells are specifically interacting with Mtb-infected AM.” However, I believe it is possible that these AM were activated by bystander IFNg in the absence of a cognate interaction, thus this statement should be softened. To make this statement the authors would need to do MHCII-/- mixed chimeras and show a difference in IFNg activated genes in MHCII+ vs. MCHII- AMs.

Although we agree that IFNγ could be activating AM in the absence of direct cognate interaction with T cells, we favor our interpretation. If IFNγ was acting through a bystander mechanism, we would have expected there to be little difference in the expression of IFNγ-induced genes between YFPpos AM vs. YFPneg AM. However, this is not what we find. Instead, we find that IFNγ is preferentially activating YFPpos AM. Therefore, we favor that this is not bystander activation but instead specific activation.

8) It is unclear how the predicted pathways were determined in Fig 4D – was this using a bioinformatics approach or purely plotting known stimuli that could be involved in activating macrophages? The graph needs to be explained more clearly. Do the bars represent relative expression or p-value?

The predicted upstream regulators were determined using the Inguenity IPA bioinformatic platform. We have now labelled it as “predicted upstream regulators”. The bars represent -log10[p-value] as indicated in the figure. This is now better described in the results, the figure legend, and in the methods.

9) In Fig 4E-G, the Ingenuity platform used to assess the transcriptional data. I found this graph confusing. I am not very familiar with this platform, and perhaps that is the main problem, but perhaps other readers will also be unfamiliar and I believe it needs more explanation. For example, does Fig. 4F indicate that there are no pathways predicted to be significantly upregulated by infection in the MDC population? This seems inconsistent with Fig4B and 4H, which show several Th1-related genes upregulated? IL1b downregulations is highlighted in the text, but IL-1a and IL-12 are both upregulated in the 4B scatter plot. Please clarify.

The Ingenuity pathway analysis uses the gene expression data to predict canonical pathways are actively regulated in the cell, and whether those pathways are being upregulated or downregulated. The graph shows 6-10 canonical pathways that are identified in each cell type. There is a “p” value associated with each pathway, as well as the fraction of genes that are actually regulated in each pathway. As with all of these types of analyses, the pathways don’t always make sense. Sometimes this is because of the name of the pathway: the neuroinflammation pathway includes Th1 genes including IL-12 and iNOS. Another example is the cell type specificity: Th1 pathway consists of genes mostly expressed in Th1 cells. Finally, a pathway can be identified by the overlap of a small number of genes. So, AM and MDC have 14 and 16 genes in common with the neuroinflammation pathway, but only 8 of those genes are in common. Whether the pathway is predicted to be up- or down-regulated is based on the Z-score and is only a prediction. In contrast, the data in Fig.5B is the actual expression data. The other important factor is that we are comparing the expression of genes in infected vs. uninfected cells. Thus, although the MDC express higher levels of NOS2, the AM have a higher fold-induction following infection. Finally, we are only listing the top pathways for each cell type. There are other pathways that are predicted, but the uncertainty associated with the prediction is greater. We have added additional details to the text and figure legends to clarify these issues.

10) In Figure 5, 25 genes were shown to be differentially expressed by AM, RM, and MDC that were infected, compared to those that were uninfected. The authors then went on to validate protein expression for three of these genes using antibodies (Abca1, CD38, and CD14) and investigate infected cells expressing these 3 markers for the rest of the paper. However, it is not clear how these 3 markers were chosen from amongst the 25, as the expression levels of these 3 genes were relatively low compared to others. Were other validations also attempted? Or were these 3 just the best antibodies? Without an explanation the designation of Triple and double positive cells in the remainder of the manuscript seems somewhat arbitrary.

In deciding which molecules to validate by flow cytometry, we selected molecules that were (1) expressed on the cell surface and (2) had commercially available reagents that we could fit into our flow panel. In addition to CD14, CD38, and Abca1, we also confirmed Podoplanin and NOS2. NOS2 was also selected because of its known functional importance to TB infection and we had prior experience detecting intracellular NOS2. We are in the process of expanding a list of markers that we can detect. Although the expression of some of the molecules appears low, most of them are highly expressed and another important factor is the expression in infected cells vs. uninfected cells.

11) The importance of the BMDC experiments in Fig8A-B is unclear, even as stated by the authors themselves, and may not be needed in the manuscript. What do the CD38-negative cells represent? Are these cells that just haven’t fully differentiated in the in vitro culture? Is the left-hand plot in Fig8A the uninfected controls?

The left-hand plot is an uninfected control and the CD14/CD38 double negative cells are mostly MHCII high cells. We agree with the reviewer that this data is not needed in the manuscript and as it is mainly negative data, we have removed Figure 8A-B and its discussion in the results section.

12) Do the authors have any insights about why, in Fig. 8D ABCA1 expression goes down with IFNg in vitro (unlike CD38 and CD14) even though it’s highly expressed in vivo?

Previous work finds that IFNγ treatment in vitro reduces Abca1 levels on macrophages. The appearance of Abca1 positive cells during TB infection indicates that some other signal, produced by the inflammatory milieu promotes Abca1 expression. For example, known regulators of ABCA1 include cholesterol and other lipids and nuclear hormone receptors. We have now cited this in the text.

13) Figure 7D depicts NOS2 expression by various myeloid populations based Boolean gating of the markers ABCA1, CD38, and CD14. A comparison of NOS2 staining in Mtb infected AM vs. MDC vs. RM would also be interested. The authors make conclusions about exposure of all of these myeloid populations to IFNg, but this conclusion is based on RNA-Seq of sorted bulk populations of infected cells. These populations may include subpopulations that were and were not exposed to IFNg. Assessing NOS2 at a single cell level would be informative, as the % positive may vary and this may mean some populations contain cells that are less exposed to IFNg than others. If so, the conclusions about the bulk population being exposed to T cells and IFNg should be softened, as there may be subpopulations, even perhaps large subpopulations that are not.

We find that the MDC population has the highest frequency of NOS2+ cells. These results not only reflect the exposure to IFNγ, but also the exposure to TLR signals. We think that the finding that the MDC have the greatest frequency of NOS2+ cells is consistent with the finding that on a per cell basis, they also have the highest frequency of Mtb-infected cells. We added the new analysis to the Result section focusing on the main cell populations AM, MDC, and RM (see new Fig.8a).

14) The authors use a YFP-expressing Mtb strain that was transformed using a plasmid that expresses YFP driven by a constitutive promoter. Usually plasmids are lost over time and at late timepoints most infected cells no longer express the plasmid and are fluorescent. Thus, the utility of these strains are usually restricted to the first few weeks of infection. In Figure 9 and 10 this strain is used to identify infected cells even 16 weeks post-infection. What percentage of the colonies continue to be fluorescent at each of these timepoints (3 weeks, 6 weeks, and 16 weeks)? (In Figure 10 there seems to be a much lower % of YFP+ cells at 10 weeks than at 6 weeks, whereas overall CFUs usually don’t drop in this timeframe, suggesting plasmid loss). Unless the authors can convincingly show that the plasmid is not preferentially lost in some cell types relative to other cell types (which may be true if the Mtb replication rate is different in different cell types), I suggest that the authors not show data at timepoints in which the % of infected cells that still express YFP is low, as it would be difficult to draw conclusions.

We did not measure systematically measure CFU in every experiment as large numbers of cells were needed for the flow cytometric analysis. Although we can detect YFP signal as late as 9 months after infection, our CFU data is consistent with considerable plasmid loss at late time points. We included this data, because the overall distribution of YFP-expressing bacilli among different cell types appears to be relatively constant. Nevertheless, we have removed the data from 16 weeks from figures 9 and 10. Instead, we have placed the data showing the full-time course in the supplemental data with the caveat raised by the reviewer (SFig.6).

15) The conclusions on p16 regarding AM, and there highly activated state should be softened considerably. In this context, the work of Rothchild et al should be recognized which shows that AM are very slow to upregulate pro-inflammatory pathways during the first 10 days of infection. It should also be acknowledged that the conclusions from bulk RNA-Seq could mean that a subset of AM are activated by IFNg, whereas other subpopulations may not. This would need to be evaluated on a single cell level, preferentially in tissue sections to identify the relative locations of activated and unactivated cells.

While it is possible that there are subpopulations of AM that are not resolved by our transcriptional analysis, our flow cytometric analysis shows that nearly all of the AM are activated based on their nearly uniform expression of CD11b and MHCII (Fig.1 and Fig.11). While this pervasive activation of AM is independent of their infection status, we find that YFP+ AM are further activated. An important difference from the study by Rothchild et al is our later time point. During the first 10 days of infection, there are few antigen-specific T cells in the lung and therefore lower amounts of IFNγ. Week 3 is near the peak of the pulmonary T cell response. Nevertheless, we now reference Rothchild et al. and elaborate on these issues.

Reviewer #3: No major revisions

**Part III – Minor Issues: Editorial and Data Presentation Modifications**

**Reviewer #1:** (No Response)

**Reviewer #2:**

1. A lot of the FACS plots are missing percentage values inside the gates (for example, Fig 2C), which would be really helpful to interpreting the data.

We have added the percentages to Fig.2c and other figures. Please note that the values are plotted in Fig.2D.

Also, in Figure 1H, should the AM percent say “85” instead of “12”?
The numbers represent the frequency of each population among the non-PMN myeloid cell, which is the parent gate in this case. We have added additional detail to the figure legend to clarify this. We also added the equivalent numbers to Fig.1I.

2. The gating strategy shown in Fig S1 does not mention the “MDC” cells and instead only refers to them as CD11b DC. While you address this later on, it would be helpful for future figures if the MDC was mentioned there.

We do refer to these cells as MDC, using a double asterisk, in the list of different cell populations. We have now tried to make this more obvious by including the asterisk in the FACS plot.

Conversely, in Fig4H you again refer to CD11b DC instead of MDC.
This has been fixed

3. The plots in Fig 4E-G should be labeled with the respective cell type for clarity.
This has been fixed

4. Please include units for the Y-axis of Fig 4H. What do the bar heights represent? Are AM showing significantly stronger expression profiles for inflammatory genes compared to MDC? If so, this would conflict with Huang et al who find that IM (MDC) are more proinflammatory relative to AM.
The axis title has now been included – the bars represent the NES (normalized enrichment score). The comparisons are not between AM vs. MDC, but rather infected AM vs uninfected AM, infected MHC vs. uninfected MDC, etc. With the uninfected cells being YFPneg cells from within the same Mtb-infected lung.

5. Is IL-1b staining shown in Figure 7 is actually pro-IL1b. If so, please label it the plots and Methods accordingly.
The reviewer is correct that our antibody only recognizes pro-IL-1b, so we have labelled the axis “pro IL-1b” and cited this in the methods.

6. Similar reporting of enriched NOS2 expression among Mtb-infected cells was reported in Huang et al and of enriched IL-1b in Cohen et al. Please acknowledge these when discussing Fig 7A. This activation status appears to be a general feature of Mtb-infected cells.

We have cited these references and Mogues et al.

**Reviewer #3:** Minor revisions and thoughts:

Are Alveolar macrophages able to degrade the YFP protein more effectively than CD11chi MDC thus leaving unstained bacteria residing in these cells? Could an acid fast stain be done on these cells to determine they have lower numbers of bacteria.

We are not aware of any measurements that have compared the ability of different cell types to degrade YFP and this would be difficult, as one would want to measure this in the infected cell, which are activated and altered metabolically. The distribution of bacteria we observe is similar to that observed by Repasy et al who used an AFB stain (PLoS Pathogens 2013). As the YFP is not secreted by the bacterium, we assume that this issue would be most relevant to the persistence of dead bacteria. An important goal for the community is developing live-dead reporters that can be used in vivo. We have tried but to date all of our constructs impaired the fitness of the bacilli.

Is there any evidence that CD11cHi MDC or equivalent cells exist in humans?

Thank you for this question. YES! This is something we should have put in the discussion. We now have included it.

The authors describe the TP CD11cHi MDC having the highest level of NOS and IL-1beta which are normally associated with an increased bacillary killing. However Mtb seems to preferentially infect and reside in these cells how is this congruent?

This is a conundrum and as we originally mentioned in the text and in response to the other reviewers, we cannot be certain whether the bacilli are viable or dead. A possibility that we favor is that NO and IL1b is associated with reduced bacillary growth (lower MFI) but not so much to sterilize the lesion. Another possibility is that some bacteria could be resistant to NO and NO could in fact adversely affect immunity to Mtb. Finally, we do we some YFPpos cells that are NOS2 negative – and these cells could be a niche for persistent bacteria. Clearly, other investigators have observed colocalization of MTb and NOS2, and yet this paradox remains.

Alterations/Additions to figures.

Figure 1I. The first panel of this subsection examining Non PMN myeloid cells is a dot plot while the rest are contour plots.

We have replaced the first panel with a contour plot.

Figure 4 Typo in legend of H

Couldn’t find typo

Figure 6 This figure lacks clarity specifically the A to H sub groups.
In Figure 7, the subgroups show in Fig.7d (i.e., A-H) are defined in Fig.7a, and also discussed in the text. We have now more explicitly refer to these populations in the Figure 7A legend.

Figure 7 Same as figure 6

In Figure 8, the subgroups shown in Fig.8d (i.e., A-H) are defined in the legend, with reference to Fig.7a. We revised the text where we discuss Fig.8d, to more explicitly refer to these populations.

Figure 8D Statistics need to be applied to these data sets.

We have included the statistical analysis for the date in Fig.9b.

Figure 8E Collated MFI for ABCA1 between WT and KO should be graphed for figure 8E

We have added the means +/- SD for the replicate values for the experiment shown in Fig.9c

Figure 10 Gates and numbers needed for this figure. Also the data should be collated and presented in this figure

We have added the means +/- SD for the replicate values for Fig.11.
